# Effect of furosemide in the treatment of high-altitude pulmonary edema

**DOI:** 10.1186/s12890-024-02933-x

**Published:** 2024-03-04

**Authors:** Dava Tenzing, Pianduo Suolang, Deji Gesang, Duoji Suolang, Gaozhan Duan, Wangmu Ciren, Yihui Wang, Tongtian Ni

**Affiliations:** 1 Department of Emergency, People’s Hospital of Shigatse City, No. 1 Daqing East Road, Sangzhuzi District, Shigatse City, Tibet China; 2 Department of Emergency, Ruijin Hospital, Shanghai Jiao Tong University School of Medicine, No. 197, Ruijin er Road, Huangpu District, 200025 Shanghai, China

**Keywords:** High-altitude pulmonary edema, Furosemide, Prognosis

## Abstract

**Background:**

High-altitude pulmonary edema (HAPE) refers to the onset of breathlessness, cough, and fever at rest after arriving at high altitudes. It is a life-threatening illness caused by rapid ascent to high altitudes. Furosemide is controversial in HAPE treatment but is routinely used in China. Further research is needed to assess its efficacy and impact on HAPE management and prognosis. The aim of this study is to determine the effectiveness of furosemide for HAPE.

**Methods:**

A retrospective was conducted to analysis of patients with HAPE admitted to the People’s Hospital of Shigatse City from January 2018 to September 2023. Patients were divided into furosemide group and non-furosemide group for further analysis. Clinical variables including demographic information, comorbidities, vital signs, inflammatory markers, biochemical analysis, CT severity score and prognostic indicators were collected.

**Results:**

A total of 273 patients were enrolled, with 209 patients in the furosemide group and 64 patients in the non-furosemide group. The furosemide group showed a significantly decrease in CT severity scores compared to the non-furosemide group. Subgroup analysis showed that the longer the duration of furosemide use, the more pronounced the improvement in lung CT severity scores. But there were no significant differences in length of hospital stay and in-hospital mortality between the two groups.

**Conclusion:**

Furosemide helps alleviate pulmonary edema in HAPE patients, but further research is needed to clarify its impact on prognosis.

**Supplementary Information:**

The online version contains supplementary material available at 10.1186/s12890-024-02933-x.

## Background

High-altitude pulmonary edema (HAPE) refers to the onset of breathlessness, cough, and fever at rest after arriving at high altitudes [1]. It is a common high-altitude illness that occurs when individuals rapidly ascend to altitudes typically above 2,500 m [2]. It is characterized by the accumulation of fluid in the lungs, leading to severe respiratory distress [3]. With the increasing popularity of high-altitude tourism and mountaineering in high-altitude regions, the overall number of HAPE cases has also been on the rise [4]. HAPE poses a significant health risk to individuals who venture into high-altitude regions.

Currently, the main treatment options for HAPE include descending to lower altitudes, supplemental oxygen, and the use of medications such as nifedipine [5]. However, these interventions may not always be readily available or effective in all cases.

Furosemide, a diuretic medication, acts by inhibiting the reabsorption of sodium and chloride in the renal tubules, resulting in increased urine output and subsequent reduction in fluid overload [6]. Its use in the treatment of HAPE is controversial, and there is limited research on this topic [6]. However, in China, furosemide is included as a routine treatment measure for HAPE [7, 8].

Therefore, it is crucial to study the efficacy of furosemide in the treatment of HAPE, as it has the potential to significantly impact the management and prognosis of HAPE patients. The aim of this study is to determine the overall effectiveness of furosemide as a therapeutic agent for HAPE.

## Methods

### Study subjects

We conducted a retrospective analysis of patients with HAPE admitted to the People’s Hospital of Shigatse City from January 2018 to September 2023. Inclusion criteria were age greater than or equal to 18 years and onset of symptoms within one week of arrival in high-altitude areas. Exclusion criteria were pregnancy or lactation, average arterial pressure < 65mmHg on admission, hospitalized for less than 3 days, and incomplete data (patients who did not undergo lung Computed Tomography (CT) assessments before and after treatment). The follow-up period extended from the time of admission to discharge. This study was approved by the Ethics Committee of the People’s Hospital of Shigatse City, and written informed consent was obtained from each patient or their family members (2023RKZRMYY12M008).

### Patient management

During the emergency department visit, all patients underwent lung CT scans to confirm the presence of pulmonary edema. Upon admission, all patients received supplemental oxygen and bed rest. Symptomatic supportive treatment, including appropriate anti-infection and antihypertensive therapy, was administered based on the presence of complications such as respiratory tract infections and hypertension. The use of diuretics was determined by the attending physician’s experience. All patients completed lung CT examination three days after treatment. Patients were divided into a furosemide group and a control group based on whether furosemide was used on the day of admission. Subgroup analysis was further conducted based on the dosage and duration of furosemide use.

### Clinical variables

Clinical variables were obtained by retrieving relevant data from the electronic medical record system and manually reviewing them, including demographic information, comorbidities, clinical assessments (vital sign), inflammatory markers (white blood cell count, C-reactive protein, procalcitonin), biochemical analysis (aspartate aminotransferase, total bilirubin, blood urea nitrogen, serum creatinine, potassium, glucose), D-dimer and arterial blood gas. The HAPE prognostic indicators recorded in this study included hospitalization duration and in-hospital mortality.

Radiographic assessment of pulmonary edema severity was performed by two experienced radiologists using the CT Severity Score. This method assigns a percentage score to pulmonary edema in each of the five lung lobes [9]. The total CT score is the sum of scores from all lobes, ranging from 0 (no involvement) to 25 (maximum involvement). The specific scoring for each lung lobe is as follows:

1 point, < 5% involvement;

2 points, 5-25% involvement;

3 points, 26-49% involvement;

4 points, 50-75% involvement;

5 points, > 75% involvement.

### Statistical analysis

Clinical data were analyzed using SPSS 26.0 statistical software. For missing values within 10% of the data, the mean substitution method is used, and for missing values exceeding 10%, the data are excluded. Independent sample t-tests or Mann-Whitney U tests were used for comparison of means, while chi-square tests or Fisher’s test were used for comparison of categorical variables. *P*-value < 0.05 was considered statistically significant.

## Results

### Clinical characteristics of the patients

**Fig. 1:**
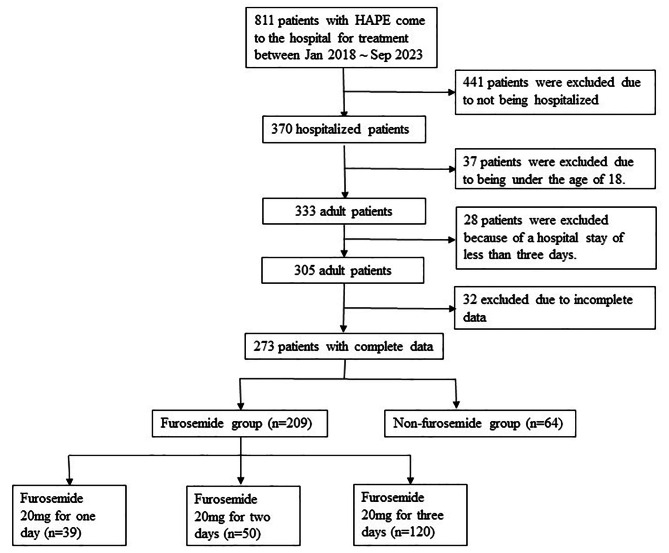
Flow chart describing the patients with high-altitude pulmonary edema between Jan 2018 ∼ Sep 2023

From January 2018 to September 2023, a total of 811 patients with HAPE visited the People’s Hospital of Shigatse City (Fig. 1). According to the inclusion criteria, a total of 273 patients were finally enrolled, with an average age of 34.15 years and a male-to-female ratio of 6:1. Among them, there were 209 patients in the furosemide group and 64 patients in the non-furosemide group. Based on the duration of furosemide use, the furosemide group was further divided into the group using furosemide on the first day after admission (F1 group)(*n* = 39), the group using furosemide for the first two days after admission (F2 group)(*n* = 50), and the group using furosemide for the first three days after admission (F3 group)(*n* = 120). In terms of baseline clinical characteristics upon admission, the heart rate of patients in the non-furosemide group was significantly higher than that of patients in the furosemide group, but their levels of procalcitonin, hematocrit, platelet count, and blood potassium were significantly lower than those in the furosemide group (*P* < 0.05). There were no significant differences in other medical history, vital signs, and laboratory indicators (Table 1).

**Table 1:** Characteristics of patients with high altitude pulmonary edema on admission (n%; x ± s; median (IQR))

	Non-furosemide group (*n*=64)	Furosemide group (*n*=209)	*P* Value
Mean age (years)	35.64 ± 10.98	33.69 ± 10.87	0.212
Gender (male%)	55.00 (85.94%)	179.00 (85.65%)	0.953
Body Mass Index (kg/m^2^)	23.08 ± 1.42	22.81 ± 1.57	0.226
Altitude before entering the high altitude area (m)	1246.00 ± 554.31	1219.65 ± 453.40	0.701
Altitude at the time of onset (m)	3728.42 ± 70.49	3737.26 ± 130.48	0.604
Time of onset after entering high altitude area (day)	4.19 ± 2.00	4.29 ± 1.94	0.708
Current smoker (%)	15.00 (23.44%)	53.00 (25.36%)	0.756
Alcohol abuse (%)	22.00 (34.38%)	73.00 (34.93%)	0.935
Past history			
Hypertension (%)	3.00 (4.69%)	7.00 (3.35%)	0.618
Diabetes mellitus (%)	2.00 (3.13%)	8.00 (3.83%)	0.793
Coronary heart disease (%)	0.00 (0.00%)	0.00 (0.00%)	/
Heart failure (%)	0.00 (0.00%)	0.00 (0.00%)	/
Cerebrovascular disease (%)	0.00 (0.00%)	1.00 (0.48%)	1.000
Peripheral vascular disease (%)	0.00 (0.00%)	1.00 (0.48%)	1.000
COPD (%)	0.00 (0.00%)	0.00 (0.00%)	/
Connective tissue disease (%)	0.00 (0.00%)	0.00 (0.00%)	/
Peptic ulcer (%)	0.00 (0.00%)	0.00 (0.00%)	/
Chronic Liver Disease (%)	0.00 (0.00%)	0.00 (0.00%)	/
Chronic renal failure (%)	0.00 (0.00%)	0.00 (0.00%)	/
Solid tumor (%)	0.00 (0.00%)	0.00 (0.00%)	/
Leukaemia (%)	0.00 (0.00%)	1.00 (0.48%)	1.000
Lymphoma (%)	0.00 (0.00%)	0.00 (0.00%)	/
HIV (%)	0.00 (0.00%)	0.00 (0.00%)	/
Vital sign			
Body temperature (℃)	36.60 ± 0.42	36.59 ± 0.48	0.918
Heart rate (beat per minute)	98.95 ± 17.54	92.86 ± 17.47	0.015*
Respiratory rate (breaths per minute)	21.23 ± 1.83	21.05 ± 3.84	0.722
Systolic pressure (mmHg)	117.02 ± 16.55	117.68 ± 17.27	0.785
Diastolic pressure (mmHg)	81.25 ± 12.94	78.92 ± 12.74	0.204
Mean arterial pressure (mmHg)	93.17 ± 12.88	91.84 ± 13.02	0.475
Laboratory index			
White blood cell (×10^9^/l)	9.51 ± 3.41	10.27 ± 4.01	0.169
C-reactive protein (µg/ml)	8.00 (8.12)	7.00 (6.50)	0.435
Procalcitonin (ng/ml)	0.24 ± 0.06	0.26 ± 0.07	0.032*
Hemoglobin (g/l)	151.88 ± 25.24	157.85 ± 22.49	0.072
Hematocrit (%)	44.95 ± 7.09	46.95 ± 6.31	0.032*
Platelet (×10^9^/l)	198.01 ± 69.28	236.57 ± 69.88	< 0.001*
Alanine aminotransferase (U/l)	34.00 (26.75)	28.00 (21.50)	0.918
Total bilirubin (µmol/l)	17.46 ± 12.35	17.96 ± 8.38	0.711
Albumin (g/l)	43.67 ± 5.29	43.80 ± 5.49	0.861
Prealbumin (g/l)	221.45 ± 68.90	213.07 ± 59.33	0.342
Serum creatinine (µmol/l)	88.68 ± 25.64	85.40 ± 26.48	0.383
Blood urea nitrogen (mmol/l)	6.32 ± 2.64	6.34 ± 2.17	0.942
Blood potassium (mmol/l)	3.57 ± 0.39	3.73 ± 0.44	0.008*
Blood glucose (mmol/l)	6.11 ± 2.59	5.82 ± 1.89	0.328
D-dimer (mg/l)	0.47 (0.38)	0.48 (0.41)	0.396
Potential of hydrogen	7.43 ± 0.04	7.42 ± 0.05	0.255
PaO2 (mmHg)	42.55 ± 6.69	42.86 ± 7.56	0.769
Oxygenation index	202.60 ± 31.84	204.08 ± 35.99	0.769

Note: COPD: Chronic obstructive pulmonary disease. HIV: Acquired Immune Deficiency Syndrome. *P < 0.05 was considered statistically signifcant

### Influence of furosemide on prognosis of patients with high-altitude pulmonary edema

There were no significant differences in CT Severity Scores between the furosemide group and the non-furosemide group before and after treatment (*P* > 0.05) (Table 2). And we did not find any differences between the two groups in terms of the rate of invasive mechanical ventilation, length of hospital stay, and in-hospital mortality (Table 3). However, by analyzing the difference in the decrease of CT Severity Scores, the furosemide group showed a significantly higher degree of decrease compared to the non-furosemide group (*P* = 0.003) (Table 2).

**Table 2:** Effect of furosemide on pulmonary CT of patients with high altitude pulmonary edema

CT severity score	Non-furosemide group (*n*=64)	Furosemide group (*n*=209)	*F*	*P* value
Before treatment	10.28 ± 4.13	11.36 ± 4.07	0.532	0.065
After treatment	4.39 ± 1.78	4.22 ± 1.99	0.438	0.539
Score difference	5.89 ± 2.99	7.14 ± 2.63	6.105	0.003*

Note: *P < 0.05 was considered statistically signifcant

**Table 3:** Effect of furosemide on prognosis of patients with high altitude pulmonary edema

	Non-furosemide group (*n*=64)	Furosemide group (*n*=209)	*P* value
IMV (%)	1 (1.56%)	4 (1.91%)	0.859
Length of stay (day)	5.85 ± 2.28	5.68 ± 2.36	0.602
In-hospital mortality (%)	1 (1.56%)	1 (0.48%)	0.415

Note: IMV: Invasive mechanical ventilation

### Subgroup analysis of the pulmonary radiographic features and length of hospital stay in patients with HAPE treated with furosemide

**Fig. 2:**
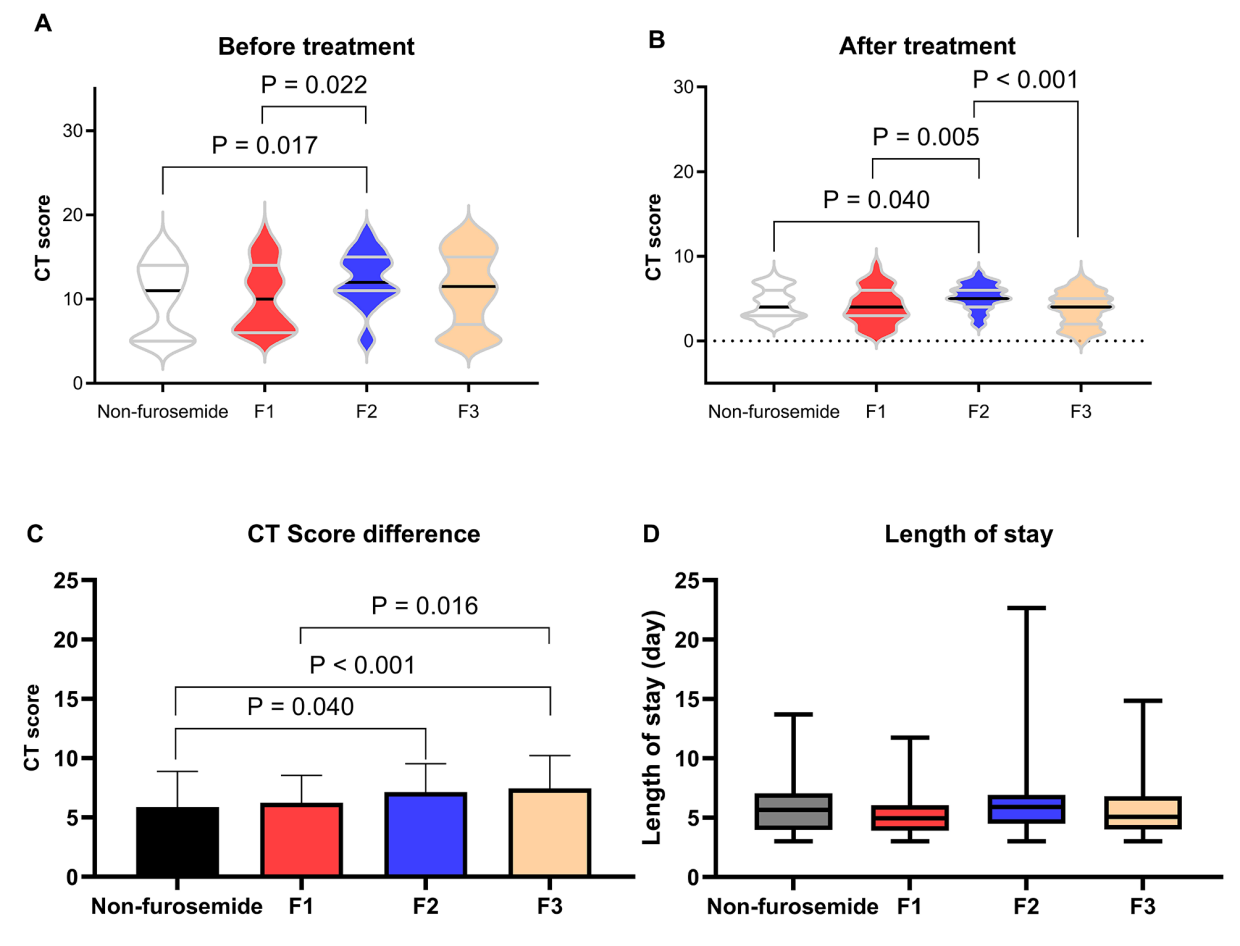
Subgroup analysis of the pulmonary radiographic features and length of hospital stay in high-altitude pulmonary edema treated with furosemide. (**A**) CT Severity Score of high-altitude pulmonary edema patients among different subgroups before treatment. (**B**) CT Severity Score of high-altitude pulmonary edema patients among different subgroups after three days of treatment. (**C**) Difference in CT Severity Score of high-altitude pulmonary edema patients among different subgroups before and after treatment. (**D**) Length of hospital stay among high-altitude pulmonary edema patients in different subgroups. F1: Furosemide 20 mg daily used on the first day after admission. F2: Furosemide 20 mg daily used for the first two days after admission. F3: Furosemide 20 mg daily used for the first three days after admission

Through subgroup analysis, it was found that before treatment, both the non-furosemide group and the F1 group had significantly lower CT Severity Scores compared to the F2 group (10.28 ± 4.13, 10.36 ± 3.94 vs. 12.40 ± 3.22) (*P* < 0.05) (Fig. 2A). After treatment, the CT Severity Scores of the non-furosemide group and the F1 group remained significantly lower than the F2 group (4.39 ± 1.78, 4.13 ± 2.13 vs. 5.26 ± 1.51) (*P* < 0.05). However, the CT Severity Scores of the F3 group showed a significant decrease compared to the F2 group (3.82 ± 1.97 vs. 5.26 ± 1.51) (Fig. 2B). By analyzing the difference in CT Severity Scores before and after treatment (Fig. 2C), it was found that the F2 group had a greater decrease in scores compared to the non-furosemide group (7.14 ± 2.39 vs. 5.89 ± 2.99) (*P* < 0.05), and the F3 group had a greater decrease in scores compared to the non-furosemide group and the F1 group (7.44 ± 2.77 vs. 5.89 ± 2.99, 6.23 ± 2.32) (*P* < 0.05). However, there were no significant differences in length of hospital stay between the subgroups (Fig. 2D).

## Discussion

The findings of this study indicate that although the use of furosemide can accelerate the absorption of lung infiltrates in patients with HAPE. However, the clinical implications of these improvements in imaging findings on prognosis are still unclear, possibly due to the limited number of cases. It does not have a significant impact on reducing the length of hospital stay and in-hospital mortality.

HAPE is a non-cardiogenic pulmonary edema and differs from cardiogenic pulmonary edema, such as congestive heart failure [10]. Cardiogenic pulmonary edema occurs when heart disease leads to a weakened pumping function of the heart or an excessive cardiac load, resulting in pulmonary congestion and increased pulmonary capillary pressure, ultimately causing fluid leakage into the lungs [11]. Diuretics are commonly used medications for treating congestive heart failure, as they promote urine excretion to reduce fluid retention in the body and alleviate pulmonary edema symptoms [12]. However, HAPE is caused by factors such as thin air at high altitudes and pulmonary vasoconstriction [13]. Pulmonary vasoconstriction is associated with increased pulmonary vascular resistance and pulmonary artery pressure in patients [14]. These changes result in an increase in pulmonary capillary hydrostatic pressure, followed by fluid leakage from the vascular space into the interstitium and alveolar spaces [15]. Diuretics have limited effectiveness in treating HAPE, possibly due to the different pathogenesis of HAPE compared to congestive heart failure, and their limited ability to improve pulmonary vasoconstriction. Furosemide, as a loop diuretic, was found in our study to accelerate the absorption of lung infiltrates.

Through the study of lung imaging, it was found that although there was no difference in the severity scores of lung CT between HAPE patients before and after treatment, there was a difference in the change in scores. Patients who received furosemide showed a more significant reduction in lung infiltrates. Subgroup analysis further revealed that the longer the duration of furosemide use, the more pronounced the improvement in lung CT scores. Previous study [16] primarily focused on the impact of diuretics on blood pressure and did not compare changes in pulmonary imaging. However, nifedipine, as one of the therapeutic drugs, may also have an effect on blood pressure [17]. Therefore, this should not be a reason to hinder the use of diuretics in HAPE.

However, we also observed that although there may be improvements in imaging, these improvements may not necessarily translate into substantial improvements in prognosis, possibly due to the limited number of cases included. In the two cases of mortality, one was complicated by high-altitude cerebral edema, while the other had a high suspicion of pulmonary embolism. It is important to note that the length of hospital stay can be influenced by various factors, including subjective symptoms reported by patients, psychological factors, and transportation issues.

It is noteworthy that the mortality rate is high in HAPE patients who do not receive effective treatment [18–20]. One of the most crucial measures for managing HAPE is to promptly descend to a lower altitude [15]. However, it is interesting to observe that all the patients included in this study chose to remain at an altitude of 3820 m in the People’s Hospital of Shigatse City for treatment, and they achieved favorable treatment outcomes. This suggests that in cases where HAPE occurs, if a standardized treatment center can be promptly accessed, the descent to a lower altitude may be delayed without compromising the effectiveness of treatment. Marieke et al [21]. reported that the majority of HAPE patients transported to the hospital fully recovered, which is consistent with our study.

The average age of the patients enrolled in this study was 34.15 years, and there was a minimal presence of patients with pre-existing chronic conditions. This could be attributed to the fact that individuals visiting high-altitude areas are aware of the potential impact on their physical health, which seems to be different from what was previously described in the literature [22] as a lack of understanding among novices regarding the hazards of high-altitude exposure. Consequently, the study population consisted of individuals who were generally in good health prior to the onset of HAPE, with even patients who had comorbidities such as diabetes and hypertension being scarce.

The average time of onset for HAPE in this study was approximately 4 days after arriving at high-altitude areas, which is consistent with the 2–5 days reported in the previous literature [23]. The latest onset of symptoms in our study occurred on the tenth day after entering the high-altitude area, which is later than what has been reported in the literature [24]. This difference may be related to variations in factors such as oxygen supply facilities, level of exertion, and individual susceptibility.

Further research with larger sample sizes and longer follow-up periods is needed to elucidate the impact of imaging improvements on clinical outcomes and to better understand the various factors influencing hospital stay duration in HAPE patients.

### Limitation

As our study is retrospective, it is prone to bias. The mortality rate of patients with HAPE after formal treatment is lower, and a larger sample size is needed to determine the effect of furosemide on in-hospital mortality in patients with HAPE.

## Conclusion

In summary, although the use of furosemide could help to improve the lung CT imaging findings in patients with HAPE, there are no significant benefits in terms of length of hospital stay and in-hospital mortality. Clinicians still need to assess the use of such medications based on individual circumstances. And we suggest that in cases where standardized treatment centers can be accessed promptly, the decision to delay descent to a lower altitude may be considered.

### Electronic supplementary material

Below is the link to the electronic supplementary material.

Supplementary Material 1

## Data Availability

The datasets used and/or analysed during the current study are available from the corresponding author on reasonable request.

## Abbreviations

HAPE: High-altitude pulmonary edema
CT: Computed Tomography

## References

[1] Clark ST, Sheraton MEMS, High-Altitude. Field Prophylaxis And Treatment. StatPearls. Treasure Island (FL) ineligible companies. Disclosure: Mack Sheraton declares no relevant financial relationships with ineligible companies.2023. 32809512

[2] Li Y, Zhang Y, Zhang Y (2018). Research advances in pathogenesis and prophylactic measures of acute high altitude illness. Respir Med.

[3] Basnyat B, Murdoch DR (2003). High-altitude illness. Lancet.

[4] Luks AM, Hackett PH (2022). Medical conditions and high-Altitude Travel. N Engl J Med.

[5] Stream JO, Grissom CK (2008). Update on high-altitude pulmonary edema: pathogenesis, prevention, and treatment. Wilderness Environ Med.

[6] Simancas-Racines D, Arevalo-Rodriguez I, Osorio D, Franco JV, Xu Y, Hidalgo R (2018). Interventions for treating acute high altitude illness. Cochrane Database Syst Rev.

[7] Zheng BH, Li SZ, He Y, Wang HB, Li SS (2007). [The effect of inhaled nitric oxide on endothelium-derived angiokinetic factors in patients with acute high altitude disease]. Zhonghua Jie He He Hu Xi Za Zhi.

[8] Wang W, Zhang X, Ma Y (1998). [Low-concentration nitrous oxide inhalation in the treatment of high-altitude pulmonary edema]. Zhonghua Jie He He Hu Xi Za Zhi.

[9] Saeed GA, Gaba W, Shah A, Al Helali AA, Raidullah E, Al Ali AB (2021). Correlation between Chest CT Severity Scores and the clinical parameters of adult patients with COVID-19 pneumonia. Radiol Res Pract.

[10] Gojkovic M, Darmasaputra GS, Velica P, Rundqvist H, Johnson RS (2020). Deregulated hypoxic response in myeloid cells: a model for high-altitude pulmonary oedema (HAPE). Acta Physiol (Oxf).

[11] Dobbe L, Rahman R, Elmassry M, Paz P, Nugent K (2019). Cardiogenic pulmonary edema. Am J Med Sci.

[12] Novak JE, Ellison DH (2022). Diuretics in States of volume overload: Core Curriculum 2022. Am J Kidney Dis.

[13] Tetzlaff K, Swenson ER, Bartsch P (2022). An update on environment-induced pulmonary edema - when the lungs leak under water and in thin air. Front Physiol.

[14] John J, Palevsky H (2011). Clinical pharmacology and efficacy of inhaled iloprost for the treatment of pulmonary arterial hypertension. Expert Rev Clin Pharmacol.

[15] Luks AM, Swenson ER (2020). COVID-19 Lung Injury and High-Altitude Pulmonary Edema. A false equation with dangerous implications. Ann Am Thorac Soc.

[16] Hultgren HN, Letter (1975). Furosemide for high altitude pulmonary edema. JAMA.

[17] Easterling T, Mundle S, Bracken H, Parvekar S, Mool S, Magee LA (2019). Oral antihypertensive regimens (nifedipine retard, labetalol, and methyldopa) for management of severe hypertension in pregnancy: an open-label, randomised controlled trial. Lancet.

[18] Korzeniewski K, Nitsch-Osuch A, Guzek A, Juszczak D (2015). High altitude pulmonary edema in mountain climbers. Respir Physiol Neurobiol.

[19] Bartsch P, Swenson ER (2013). Clinical practice: Acute high-altitude illnesses. N Engl J Med.

[20] Netzer N, Strohl K, Faulhaber M, Gatterer H, Burtscher M (2013). Hypoxia-related altitude illnesses. J Travel Med.

[21] Dekker MCJ, Mremi A, Kilonzo KG, Nyakunga G, Sakita F, Mvungi M (2021). Altitude-Related disorders on Mount Kilimanjaro, Tanzania: two-year survey in a local Referral Center. Wilderness Environ Med.

[22] Berendsen RR, Bartsch P, Basnyat B, Berger MM, Hackett P, Luks AM (2022). Strengthening Altitude Knowledge: a Delphi Study to define Minimum Knowledge of Altitude Illness for laypersons traveling to high Altitude. High Alt Med Biol.

[23] Bartsch P (1999). High altitude pulmonary edema. Med Sci Sports Exerc.

[24] Bhattarai A, Acharya S, Yadav JK, Wilkes M (2019). Delayed-Onset High Altitude Pulmonary Edema: a Case Report. Wilderness Environ Med.

